# A haplotype-resolved, chromosome-scale genome assembly for the southern live oak, *Quercus virginiana*

**DOI:** 10.1093/g3journal/jkag023

**Published:** 2026-02-02

**Authors:** Laramie Aközbek, Zachary Meharg, Jillian Abendroth-McGhee, Tosin Akinsipe, Rijan Dhakal, Nicholas Gladstone, Zahida Pervaiz, Sejal Patel, Giovani Rossi, Claudia Ann Rutland, Caroline Bendickson, Adam Kranz, Ellen O Martinson, Scott P Egan, F Alex Feltus, David J Clarke, John T Lovell, Jenell Webber, Lori Beth Boston, Haley Hale, Hannah McCoy, Jane Grimwood, Sarah B Carey, Leslie Goertzen, Alex Harkess

**Affiliations:** Department of Crop, Soil, and Environmental Sciences, Auburn University, Auburn, AL 36849, United States; HudsonAlpha Institute for Biotechnology, Huntsville, AL 35806, United States; Department of Crop, Soil, and Environmental Sciences, Auburn University, Auburn, AL 36849, United States; HudsonAlpha Institute for Biotechnology, Huntsville, AL 35806, United States; Department of Crop, Soil, and Environmental Sciences, Auburn University, Auburn, AL 36849, United States; Department of Biological Sciences, Auburn University, Auburn, AL 36849, United States; Department of Biological Sciences, University of Alabama in Huntsville, Huntsville, AL 35899, United States; School of Fisheries, Aquaculture, and Aquatic Sciences, Auburn University, Auburn, AL 36849, United States; Alabama Department of Agriculture and Industries, Montgomery, AL 36107, United States; Department of Crop, Soil, and Environmental Sciences, Auburn University, Auburn, AL 36849, United States; Department of Biological Sciences, Auburn University, Auburn, AL 36849, United States; Department of Biological and Analytical Sciences, Savannah River National Lab, Aiken, SC 29808, United States; HudsonAlpha Institute for Biotechnology, Huntsville, AL 35806, United States; Department of Biological Sciences, University of Alabama in Huntsville, Huntsville, AL 35899, United States; Department of Biology, University of New Mexico, Albuquerque, NM 87106, United States; Department of Biology, University of New Mexico, Albuquerque, NM 87106, United States; Department of Biosciences, Rice University, Houston, TX 77005, United States; Praxis AI LLC, Clemson, SC 29631, United States; Praxis AI LLC, Clemson, SC 29631, United States; HudsonAlpha Institute for Biotechnology, Huntsville, AL 35806, United States; US Department of Energy Joint Genome Institute, Lawrence Berkeley National Laboratory, Berkeley, CA 94720, United States; HudsonAlpha Institute for Biotechnology, Huntsville, AL 35806, United States; HudsonAlpha Institute for Biotechnology, Huntsville, AL 35806, United States; HudsonAlpha Institute for Biotechnology, Huntsville, AL 35806, United States; HudsonAlpha Institute for Biotechnology, Huntsville, AL 35806, United States; HudsonAlpha Institute for Biotechnology, Huntsville, AL 35806, United States; US Department of Energy Joint Genome Institute, Lawrence Berkeley National Laboratory, Berkeley, CA 94720, United States; HudsonAlpha Institute for Biotechnology, Huntsville, AL 35806, United States; Department of Biological Sciences, University of Alabama in Huntsville, Huntsville, AL 35899, United States; HudsonAlpha Institute for Biotechnology, Huntsville, AL 35806, United States

**Keywords:** *Quercus virginiana*, live oak, syngameon, genome assembly

## Abstract

Hybridization is a major force driving diversification, migration, and adaptation in *Quercus* species. While population genetics and phylogenetics have traditionally been used for studying these processes, advances in sequencing technology now enable us to incorporate comparative and pan-genomic approaches as well. Here, we present a highly contiguous, chromosome-scale and haplotype-resolved genome assembly for the southern live oak, *Quercus virginiana*, the first reference genome for section *Virentes*, as part of the American Campus Tree Genomes program. Originating from a clone of Auburn University's historic “Toomer's Oak,” this assembly contributes to the pool of genomic resources for investigating recombination, haplotype variation, and structural genomic changes influencing hybridization potential in this clade and across *Quercus*. It also provides insights into the architecture of the putative centromeric regions within the genus. Alongside other oak references, the *Q. virginiana* genome will support research into the evolution and adaptation of the *Quercus* genus.

## Teaser

Aközbek et al. present a chromosome-scale genome assembly for southern live oak (*Quercus virginiana*), the first reference for section *Virentes*. This reference enables other researchers studying *Quercus* evolution and genetics to conduct further comparative genomics and population genetic studies. Comparative analyses within the genus reinforce previous research that the major oak sections are highly syntenic. Additionally, this assembly provides a detailed characterization of putative centromeric regions in *Quercus*, revealing a “patchwork” satellite architecture that warrants functional investigation. This assembly adds to the growing resources available in oak genomics, thus improving our ability to investigate evolutionary dynamics, adaptation, and hybridization potential within *Quercus*.

## Introduction

Hybridization has long been recognized as a powerful evolutionary force in oaks (*Quercus* spp.) that has fueled their diversification and migration across the globe as well as their adaptation to new environments (Kremer and Hipp 2020). A high potential for interfertility allows oaks to form syngameons, which are systems where 3 or more interbreeding species living in sympatry are able to retain their distinctiveness despite repeated interspecific hybridization and introgression. Although suspected to occur in other sections of *Quercus*, the syngameons of subg. *Quercus* sec. *Quercus* (∼150 species) are the most well-studied. The Southern Live Oak (*Quercus virginiana* Mill.) is a member of subg. *Quercus* sec. *Virentes* (7 species), which is sister to sec. *Quercus*, and is narrowly distributed in both North America and Cuba (Cavender-Bares et al. 2015). Members of this small section have a strong history of within-section hybridization, but are not frequently sympatric when compared with more species-rich sections. As a result, the species in sec. *Virentes* are not considered to be with a syngameon, but their participation in one still remains an open question (Cavender-Bares et al. 2015; Eaton et al. 2015; Cannon et al. 2024). The existence of natural and artificial hybrids between sec. *Quercus* and sec. *Virentes* (i.e. Compton's Oak, *Quercus × harbisonii*, etc.) indicate that reproductive barriers are minimal between them (International Oak Society, Date Unknown; Nesom 2018).

The propensity for hybridization across the genus has driven researchers to utilize population genetics and phylogenetics to examine how gene flow influences the population structure of oaks and other species, shapes their evolutionary history, and enhances their adaptive potential (Eaton et al. 2015; McVay et al. 2017; Cavender-Bares 2019; Whittemore and Miller 2023). However, a comprehensive characterization of the oak syngameon requires the synthesis of population genetic and genomic approaches (Cannon and Petit 2020). High-quality reference genomes and pan-genomes are essential to understanding this phenomenon, allowing for a more accurate and precise description of genetic variation in target species. As genomic architecture plays a critical role in the maintenance of interfertility and extensive chromosomal rearrangements between species can impact hybridization potential, comparative genomics can help reveal the genetic mechanisms underlying reproductive barriers, the extent of structural variation between and within species, the impact of this variation on gene flow, and the evolutionary dynamics shaping species divergence (Rieseberg 2001).

To help address these broad questions, we present a chromosome-scale, haplotype-resolved diploid assembly for *Q. virginiana*, a species of cultural and ecological importance in the southeastern United States. This genome was generated as part of the American Campus Tree Genomes (ACTG) program (www.hudsonalpha.org/actg), where undergraduate and graduate students assemble, annotate, and publish tree genomes from their college campuses. This *Q. virginiana* accession is a propagated clone of “Toomer's Oak,” an important symbol of Auburn University that was tragically poisoned by the herbicide Spike 80DF (Tebuthiuron) in 2010. Phylogenetically, this assembly is the first representative genome of the oak section *Virentes*. As such, it contributes to the growing genomic resources for *Quercus*. Reference genomes like the one we report here support and enrich population genetic research, as the variation between haplotypes allows researchers to identify hundreds, often thousands, of loci with distinct histories. Haplotype-resolved genomes also allow for rigorous investigation into macrostructural and microstructural variation between haplotypes within and between species. Since structural changes, such as inversions, translocations, and deletions, are suspected to play key roles in speciation, understanding these elements of the *Q. virginiana* genome will give insights into future investigations of genome evolution and species boundaries in sec. *Virentes* (Berdan et al. 2024). As more high-quality assemblies become available for highly heterozygous, sympatric *Quercus* species, they will further contribute to our understanding of the genetic basis of hybridization dynamics and the syngameons of this genus.

## Methods

### Sample collection, extraction, and sequencing

To generate whole genome sequencing data to assess the heterozygosity and genome size of *Q. virginiana*, a standard CTAB method was used to isolate DNA from young leaf tissue. 3 micrograms of input DNA was used to construct Illumina TruSeq DNA PCR-free libraries and these libraries were subsequently sequenced on an Illumina NovaSeq6000 using PE150 reads. Approximately 20 g of young leaf tissue was collected from a Toomer's Oak clone and flash-frozen in liquid nitrogen for use in PacBio HiFi sequencing. A voucher is available at the Auburn University John D. Freeman Herbarium (AUA:71025). Using the Circulomics Nanobind Plant Nuclei Big DNA kit, high molecular weight DNA was isolated from young leaf tissue with 4 g of input tissue and a 2-h lysis. The high molecular weight DNA quality was assessed via spectrophotometry for purity, via the Qubit dsDNA Broad Range assay for concentration, and run on the Agilent Femto Pulse to check fragment size. A Diagenode Megaruptor was used to shear the DNA, which was then size-selected to roughly 25 kb on a BluePippin. The SMRTbell Express Template Prep Kit 2.0 was used to build the PacBio libraries with CCS (HiFi) reads generated using 2 PacBio Sequel-II 8 M flow cells at the HudsonAlpha Genome Sequencing Center. A Dovetail Omni-C library was generated using 1 g of flash-frozen tissue as input, following the manufacturer's protocol, and sequenced on an Illumina NovaSeq6000 using PE150 reads. RNA was isolated from 4 vegetative tissue types (young leaves, brown and green senescing leaves and roots) using a modified CTAB approach followed by clean-up with a Zymo RNA Clean and Concentrator kit. RNA-seq libraries were generated using the Illumina TruSeq stranded mRNA kit following the manufacturer's protocol, and sequenced on an Illumina NovaSeq6000 using PE150 reads.

### Genome assembly and scaffolding

HiFi read quality and distribution were assessed with *nanoplot* (v1.42.0) (De Coster et al. 2018). Ploidy and heterozygosity were estimated utilizing *GenomeScope2* (v1.0.0) and *Smudgeplot* (v0.2.5) (Ranallo-Benavidez et al. 2020). The raw HiFi reads were assembled into contigs using *hifiasm* (v0.20.0-r639) with Omni-C integration (Cheng et al. 2021). The resulting haplotypes were then combined and polished with the HiFi long-reads using *racon* (v1.5.0) (Vaser et al. 2017). The assemblies were subsequently screened for contaminants with *FCS-GX* (v0.5.4), then *bbmap* (v39.13) was used to drop any contigs <25 kbp from the assembly to minimize scaffolding artifacts (Bushnell 2014; Astashyn et al. 2024). Prior to scaffolding the genome of *Q. virginiana*, we mapped the Omni-C reads to the preliminary assembly with *bwa mem* (v0.7.17, flags: -5SP -T0), then filtered for duplicate and unmapped reads with *pairtools* (v0.3.0) using default parameters (Li and Durbin 2009; Open2C et al. 2024). We scaffolded the assembly with *Yet another Hi-C Scaffolding Tool* (*YaHS*, v1.1) using default parameters (Zhou et al. 2023). We visualized and manually curated the assembly with Juicebox (v2.13.07) (Durand et al. 2016; Supplementary Fig. 1). Telomeres were identified with *GENESPACE* (v1.3.1) to check for their presence or absence as well as to assess their proper orientation within the chromosome (Lovell et al. 2022). Genome completeness and quality metrics were assessed utilizing *assemblathon2*, *merqury* (v1.3), and *compleasm* (v0.2.6) with the lineage *eudicots_odb10* (Bradnam et al. 2013; Rhie et al. 2020; Huang and Li 2023). The *Q. virginiana* assemblies were reordered and named according to the previously published *Quercus* references (Kapoor et al. 2023). The plastid genomes were assembled from the raw HiFi reads with *OatK* (v1.0), annotated with *GeSeq* (v2.0.3), and visualized with *OGDRAW* (v1.3.1) (Tillich et al. 2017; Greiner et al. 2019 ; Zhou et al. 2025).

### Genome annotations

We ran *RepeatModeler2* (v2.0.6) with the *LTRStruct* parameter to generate a de novo repeat library for each haplotype, which was used to softmask the assembly with *RepeatMasker* (v4.1.5) (Chen 2004; Flynn et al. 2020). Repetitive elements were further annotated with *EDTA* using default parameters (v2.1.3) to examine the repeat landscape (Ou et al. 2019; Supplementary Fig. 2). Centromeric monomers were identified with *TRASH* (v1) and centromeric arrays were visualized with *StainedGlass* (v0.6), *RepeatOBserverV1*, and *karyoploteR* (v1.28.0) (Benson 1999; Gel and Serra 2017; Vollger et al. 2022; Wlodzimierz et al. 2023; Elphinstone et al. 2025). Additional information regarding the identification, characterization and visualization of the *Q. virginiana* centromeres can be found in Supplementary Methods 1 in the Supplementary Material.

We performed a gene annotation with *Braker3* (v3.0.6), utilizing proteins from other *Quercus* and Fagales species as well as RNA-seq evidence from various *Q. virginiana* tissue types: young leaves, brown and green senescing leaves and roots (Gabriel et al. 2024; Supplementary Table 1). Annotations without full or partial support from RNA-seq evidence were removed from the final gene set utilizing the *Braker3* supplementary script *selectSupportedSubsets.py* to avoid inflating gene counts with unsupported, low-confidence predictions that may include monoexonic, pseudogenized or unmasked TE models. The functional annotation was performed with *EnTAP* (v2.1.0) using default parameters and the UniProt Swiss-Prot, UniProt Trembl, NCBI Refseq Plant, and NCBI NR databases. The completeness of the annotation was assessed with *BUSCO* (v5.7.0) using the *eudicots_odb10* database (Hart et al. 2020; Manni et al. 2021).

### Structural and comparative genome analyses

The haplotypes were compared with each other with *nucmer* (v4.0.0rc1) and visualized with dot (Sandbox Bio, Date Unknown; Marçais et al. 2018; Supplementary Fig. 3). Structural variants between the haplotypes and other genome assemblies were called with *syri* (v1.7.0) and *plotsr* (v1.1.0) using default parameters (Goel et al. 2019; Goel and Schneeberger 2022). *GENESPACE* (v.1.3.0) was used to build riparian plots to visualize syntenic relationships between the haplotypes as well as other *Quercus* species (Lovell et al. 2022).

## Results and discussion

**Fig. 1.**
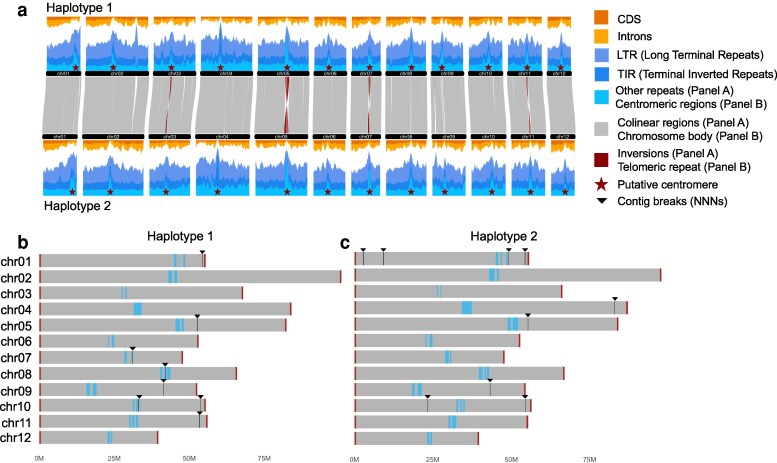
The genomic structure of *Q. virginiana*. a) The gene and repeat landscapes of both haplotypes generated by GENESPACE (Lovell et al. 2022). These landscapes are stacked bar plots where the values represent the percent of sequence attributed to each feature category at the midpoint of the sliding window (1 Mb size and 100 kb step). Although the trend is not as pronounced in some other angiosperms, repeat density tends to increase and gene density tends to decrease as both approaches the centromeres. b) A graphical representation of the *Q. virginiana* karyotype for both haplotypes, showing the proximity of breaks in contiguity to the putative centromeric satellites as well as the presence of canonical telomeric sequences flanking all chromosomes.

We present a chromosome-scale assembly for the diploid *Q. virginiana* “Toomer's Oak” using a combination of PacBio HiFi reads and Dovetail Omni-C (Supplementary Fig. 4). Nearly 200 × coverage of paired-end 150 bp Illumina sequencing data were generated, and *k*-mer frequencies were used to estimate a haploid genome size of 719 Mb and 1.57% heterozygosity (Supplementary Figs. 5 and 6 and Supplementary Table 2). Approximately 66 Gb of raw PacBio HiFi data were generated, amounting to ∼84 × estimated coverage of the haploid nuclear genome size (Supplementary Table 2 and Supplementary Fig. 7). We assessed the quality of our HiFi reads using *nanoplot*, indicating high-quality libraries and a mean read length distribution centered around 14.5 kb (Supplementary Fig. 8 and Supplementary Tables 2 and 3). Of the ∼792 M Omni-C read pairs generated scaffolding, there was a low duplication rate (Supplementary Table 4). Haplotype 1 was 788.8 Mb in length with a contig N50 of 53 Mb, and Haplotype 2 was 781.7 Mb in length with a contig N50 of 55.4 Mb (Table 1; Supplementary Table 2). In both haplotypes, ∼50% of chromosomes are contained in single contigs, and all chromosomes are flanked with canonical telomeric sequences (Table 1 and Fig. 1b; Supplementary Fig. 9). Assembly BUSCO scores are strong for both haplotypes, with ∼99% complete genes recovered (Table 1; Supplementary Table 2). *k*-mer-based completeness statistics indicate a high consensus quality score (QV > 43) and *k*-mer completeness score (98.1%) for the combined haplotypes (Table 1; Supplementary Table 2). In heterozygous species such as *Quercus*, it is not unusual for 1 haplotype to have *k*-mers from the original read set not found in the other haplotype. As a result, the *k*-mer completeness scores for the individual haplotypes (∼79% to 80%) are lower than when combined. The final assembly is haplotype-resolved with 12 chromosomes per haplotype. Structural variation between the haplotypes, as characterized by *syri*, consists primarily of 54 inversions, approximately 244 indels and 3.29 M SNPs, as well as a varying number of small duplications and translocations (Supplementary Table 5). We did not find any known contaminants, and furthermore, we searched for evidence of horizontal gene transfer (HGT) from the cynipid gall wasp *Belonocnema kinseyi*, a gall-inducing wasp hosted by *Q. virginiana*, as well as bacterial and fungal sources into the *Q. virginiana* genome, but did not find any evidence for HGT between the oak and these symbionts (Supplementary Methods S2). We also assembled and annotated the chloroplast genome of *Q. virginiana* (Supplementary Fig. 10). The size of the chloroplast genome (161 kb) is similar to that of previously published assemblies with 80 protein-coding, 30 tRNA and 4 rRNA annotations (Yang et al. 2016; Hu et al. 2019; Zhang et al. 2020).

**Table 1.** Genome assembly and annotation statistics.

Assembly		
Assembly statistics	Haplotype 1	Haplotype 2
Total contig length (Mb)	788.8	781.7
Number of chromosomes	12	12
Number of contigs	601	323
Longest contig (Mb)	100.62	97.96
Min. number of contigs containing half of assembly, L50	6	6
Shortest contig from L50 set, N50 (Mb)	53.03	55.37
Number of scaffolds	586	309
Min. number of scaffolds containing half of assembly, L50	5	5
Shortest scaffold from L50 set, N50 (Mb)	65.99	66.33
Base pair QV	43.754	43.9434
	Both = 43.8473	
*k*-mer completeness (%)	79.34	79.71
	Both = 98.08	
**Assembly BUSCO completeness (%)**		
Complete (S + D)	99.78	99.78
Single-copy (S)	96.73	96.9
Duplicated (D)	3.05	2.88
Fragmented (F)	0.09	0.09
Missing (M)	0.13	0.13
**Repeats (RepeatMasker)**		
LAI (LTR assembly index)	20.61	21.16
Repeats (%)	61.21	61.17
Annotation		
**Annotation statistics**		
Number of protein-coding genes	30,390	31,361
Number of protein-coding transcripts	35,620	36,559
Annotation BUSCO completeness (%)		
Complete (S + D)	97.6	97.2
Single-copy (S)	94.2	93.8
Duplicated (D)	3.4	3.4
Fragmented (F)	0.5	0.5
Missing (M)	1.9	2.3

After filtering, Haplotype 1 contains 30,390 predicted protein-coding genes, while Haplotype 2 contains 31,361 with BUSCO completeness scores of 97.6% and 97.2%, respectively. Approximately 81% of protein-coding genes are functionally annotated (Supplementary Table 2). The repetitive content of both haplotypes is comparable, with approximately 61% of each haplotype annotated as repetitive (Table 1; Supplementary Table 2). Retrotransposons account for 24.89% of Haplotype 1 and 24.93% of Haplotype 2 while DNA transposons represent 19.84% and 20.8% of each haplotype, respectively (Supplementary Table 2). As seen in many other angiosperm genome assemblies, gene density decreases and repeat density increases as both approaches the putative centromeres (Fig. 1a). The major structural variants found between the haplotypes are inversions, particularly at the putative centromeric locations of Chr03, Chr05, Chr07, and Chr11 (Fig. 1a).

### Comparative genomics

Numerous reference genomes for diverse *Quercus* species have been published in recent years, presenting an exciting opportunity to explore the genomic basis of reproduction in the genus using comparative genomics. The genome of *Q. virginiana* is approximately 780 Mb in size, which is close to the average genome size of other published *Quercus* assemblies: 818 Mb (Plomion et al. 2018; Ai et al. 2022; Han et al. 2022; Sork et al. 2022; Zhou et al. 2022; Kapoor et al. 2023; Rey et al. 2023; Wang et al. 2023; Luo et al. 2024; Mead et al. 2024; Larson et al. 2025; Supplementary Table 6). Alongside *Quercus variabilis*, it represents one of the most contiguous and complete assemblies within the genus, exhibiting some of the highest assembly BUSCO completeness scores reported to date (Supplementary Table 6). While its repeat content is comparable to that of more recent assemblies, the total number of predicted protein-coding genes falls on the lower end of the range observed in other *Quercus* genomes, likely due to stringent filtering criteria applied during annotation and limited sampling of diverse tissues (Supplementary Table 6).

### Centromere positioning

**Fig. 2.**
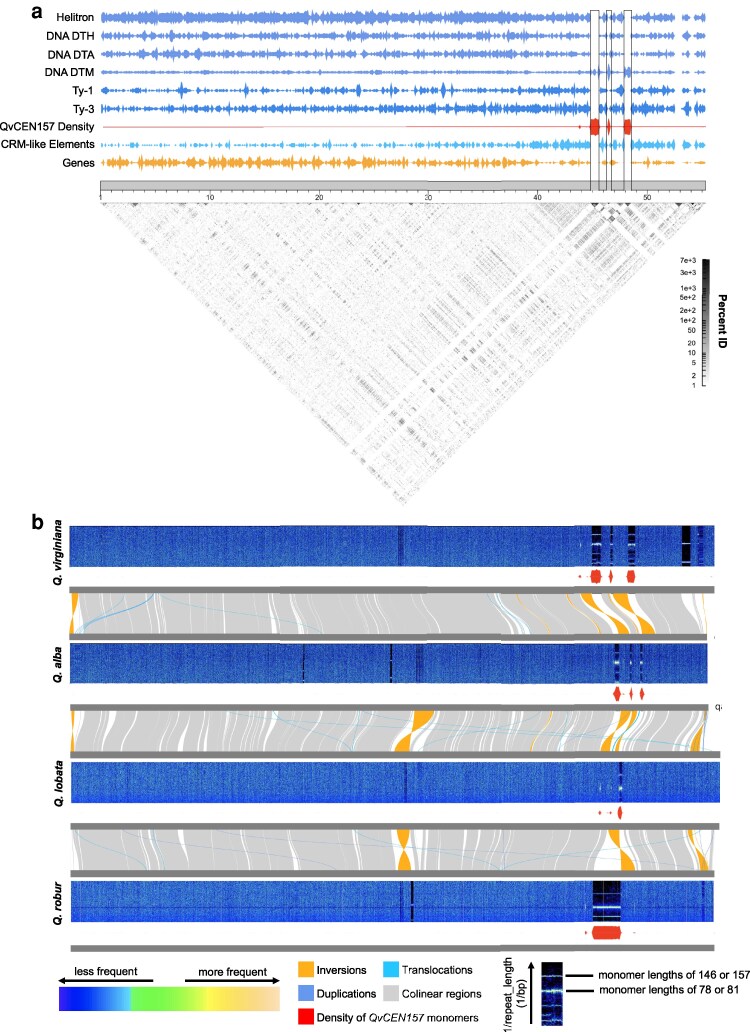
Putative centromeric satellites in chromosome 1 of *Q. virginiana* and other North American and Eurasian sec. *Quercus* species. a) The relative densities of diverse genomic features, including DNA and RNA transposable elements (blue), Helitrons (blue), genes (orange), and the *QvCEN157* monomer (red) with an identity heatmap (gray) generated with StainedGlass (with a scale bar indicating percent identity) visualized in relation to the predicted centromeric satellites. DNA-DTM, Mutator-type DNA transposon; DNA-DTH, PIF Harbinger-type DNA transposon; DNA-DTA, hAT-type DNA transposon; Ty-1, *Copia*-type retrotransposon; Ty-3, *Gypsy*-type retrotransposon. b) Chromosome-scale Fourier spectra of Chromosome 1 across diverse *Quercus* species generated by RepeatOBserver combined with a riparian plot showing base pair synteny between species as well as the density of *QvCEN157* monomers along each chromosome. The *x* axis of the spectra indicates genomic position and the *y* axis of the spectra represents the frequency of a particular repeat and the corresponding color intensity showing the abundance of the repeat at that frequency in that position of the genome. The uppermost bar is described as the true or “fundamental” frequency, and lower bars represent the harmonics of that true frequency. The top bar across these species either corresponds to 1/146 or 1/156:1/157, which agrees with the repeat monomers identified in *Q. virginiana* by TRASH. The presence of transposons can appear as “blurs” or “streaks” within and between the bars in the predicted array.

Centromere position and structure may influence recombination dynamics in plants, potentially impacting adaptation, introgression, and other evolutionary processes in a clade (Garg et al. 2024). An analysis of the putative centromeres in *Q. virginiana* suggests that Chromosome 1 is acrocentric—a trait likely shared with other oak species such as *Quercus lobata* (Sork et al. 2022; Figs. 1 and  2). The remaining chromosomes, however, exhibit varying degrees of metacentricity (Supplementary Figs. 11 and 12). Satellite sequences associated with these centromeres display a “patchwork” distribution across certain chromosomes, flanking genic regions (Fig. 2; Supplementary Figs. 11 and 12 and Supplementary Table 7). This pattern is unlikely to be the result of assembly artifacts, since only 3 of the 24 identified centromeres correspond with breaks in contiguity (Fig. 1b) and similar forms appear in other highly contiguous *Quercus* genomes despite extensive structural rearrangements in the region (Fig. 2b; Supplementary Fig. 13). It is notable that over 11.285 Mb of sparsely genic sequence, containing approximately 234 genes, resides within these regions on Haplotype 1 of *Q. virginiana*, and 210 genes can be found in the 9.368 Mb of intersatellite sequence of Haplotype 2 (Supplementary Tables 8 and 9).

This patchwork arrangement on Chr1 is also observed in other North American section *Quercus* species, such as *Q. lobata* and the recently assembled *Quercus alba*, but not in the Eurasian white oak, *Q. robur* (known as the “pedunculate oak” or “English oak”), where a single satellite is present on all chromosomes (Wellcome Sanger Institute 2022; Fig. 2b; Supplementary Fig. 13). These putative centromeric satellites in *Q. virginiana* are associated with 2 closely related monomers: *QvCEN157* and *QvCEN146* (Supplementary Fig. 14). *QvCEN157* is associated most with chromosomes 1, 2, 3, 4, 5, 6, 7, 9, and 11, whereas *QvCEN146* is found mainly on chromosomes 8, 10, and 12 (Supplementary Fig. 15). These monomer sizes are close to the reported putative centromeric monomer sizes in other oaks, such as *Q. lobata* (146 bp) and *Q. robur* (146 bp), although unsurprisingly, their sequence compositions differ (Sork et al. 2022; Xian et al. 2025; Supplementary Fig. 16). Despite this, the *Q. virginiana QvCEN157* sequence can still be used to identify putative centromeric arrays in other species via a permissive *BLASTn* search (Camacho et al. 2009; Fig. 2b).

In *Q. virginiana*, a group of DNA transposons known as Mutator elements (DNA/DTM) have invaded the putative centromeric satellites to varying degrees, particularly in those of chromosome 4 (Fig. 2a; Supplementary Fig. 17). Centrophilic transposable elements have been documented in plants; however, they are typically retrotransposons (Naish and Henderson 2024). Centrophilic DNA transposons, which propagate without an RNA intermediate, are comparatively under-represented in the literature. Mutator elements are well known for their mutagenic capabilities and are frequently linked to reproductive processes in plants (Ma and Li 2018; Dupeyron et al. 2019; Huang et al. 2024). This includes *Mutator-like element* (*MULE*) transposase-derived transcription factors such as *FAR1* (*FAR-RED IMPAIRED RESPONSE 1*) and *FSR (FAR1-RELATED SEQUENCE)*, the latter of which is found within the sparsely genic regions of the satellites on Chr1 in *Q. virginiana* (Supplementary Tables 8 and 9).

Although the multisatellite patchwork pattern is especially pronounced on chromosome 1 of this species and other taxa, the remaining chromosomes in the *Q. virginiana* genome also exhibit multiple putative centromeric satellites as do other *Quercus* lineages (Supplementary Fig. 12). However, some of these other lineages, such as *Q. robur*, do not exhibit multiple satellites. The architecture described in *Q. virginiana* and other species is notable because it has not been well-described in the literature beyond a handful of recently assembled genomes, though it may be more widespread than currently recognized (Liu et al. 2023; Gao et al. 2024). Due to the presence of interspersed genic regions, this pattern does not conform to the classic monocentric model found in many angiosperms (Naish 2024). If CENH3 (Centromere-specific Histone H3) is associated with more than 1 of these satellites, suggesting dicentricity or tricentricity, it is possible that proximity to each other enhances the stability of these regions, allowing them to behave similarly to a monocentromere, as in the stable dicentric rice centromere (Wang et al. 2013; Cuacos et al. 2015). Without additional experimental evidence, we also cannot classify these chromosomes as metapolycentric, a phenomenon observed in legumes such as *Pisum*, where multiple centromeric chromatin domains can be found across a primary constriction (Macas et al. 2023; Naish and Henderson 2024).

Recent research in *Arabidopsis* has revealed significant centromeric structural diversity both within and between species with some lineages undergoing substantial expansions of their centromeric satellites, yet CENH3 consistently localizes to only 1 to 2 Mb of centromeric regions, regardless of the array's size or composition (Naish 2024). This suggests that CENH3 localization is tightly regulated and reinforces the idea that centromere identity is epigenetically defined rather than dictated by genomic architecture. Given that CENH3 may localize to all, some, or none of these satellite sequences in *Q. virginiana* and other oaks, further experimental validation is necessary to characterize these regions. Continued advances in sequencing technology, particularly improvements in read length, accuracy, and assembly algorithms, will provide deeper insights into *Quercus* centromere evolution. As more genomes are assembled, we will have greater insights into the relationship between the evolution of these species and their centromeric architectures.

### Conserved gene order across Quercus

**Fig. 3.**
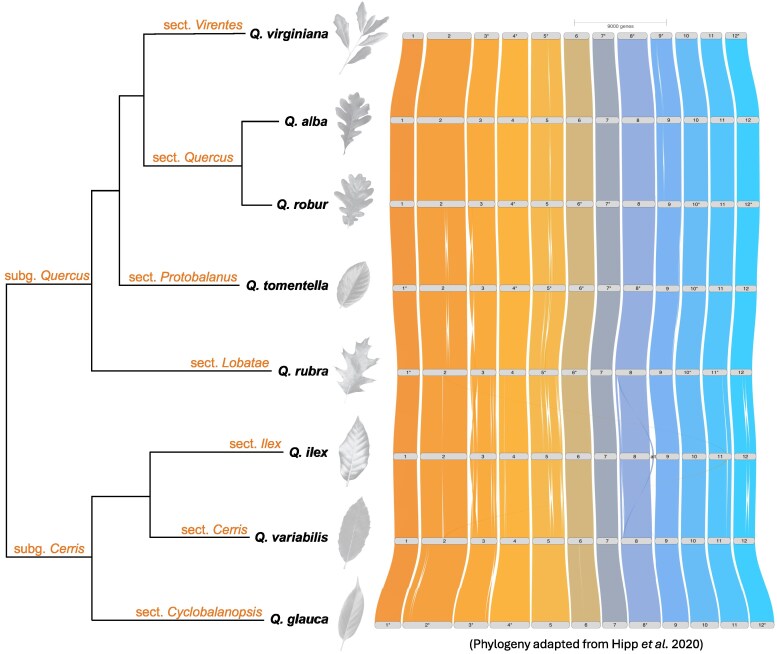
Riparian plot demonstrating conserved gene order between representative genomes from major sections of the genus *Quercus*, including *Q. alba* (Larson et al. 2025) and *Q. robur* for section *Quercus* (Wellcome Sanger Institute 2022), *Q. tomentella* (Mead et al. 2024) for section *Protobalanus*, *Q. rubra* (Kapoor et al. 2023) for section *Lobatae*, *Q. ilex* subsp. *ballota* (Rey et al. 2023) for section *Ilex*, *Q. variabilis* (Han et al. 2022) for section *Cerri*s, and *Q. glauca* (Luo et al. 2024) for section *Cyclobalanopsis*. Asterisks (*) indicate chromosomes that have been inverted relative to their original orientation for ease of visualization. Entire chromosomes are frequently oriented differently relative to each other in different genome assemblies. This does not reflect a biological phenomenon, but rather a technical artifact. The colors of the syntenic bands between chromosomes do not indicate any biological feature. The corresponding cladogram was adapted from (Hipp et al. 2020).

Additional comparative analyses between *Q. virginiana* and other *Quercus* species reveal a striking degree of synteny across divergent lineages, particularly between members of sec. *Quercus* and sec. *Virentes* (Fig. 3). At this point in time, the only remaining clade lacking a representative genome is sec. *Ponticae*, but it is likely that it is also highly syntenic with the other sections. Larger structural variants are predominantly located near putative centromeric regions or at the distal ends of chromosome arms (Fig. 3). The apparent synteny observed among *Quercus* species has been hypothesized to be both a cause and a consequence of the syngameon as conserved gene order may facilitate hybridization by reducing the likelihood of chromosomal incompatibilities, since extensive structural rearrangements are known to hinder gene flow between related taxa (Rieseberg 2001; Rieseberg 2001; Hipp et al. 2019; Cannon and Petit 2020). However, this observed conservation on a global scale may not fully capture the extent of genomic diversity between or within species, such as smaller rearrangements and translocations of TEs that may affect hybridization potential and success. To better understand the diversity of genomic architecture and its role in shaping the evolutionary dynamics within the genus, comprehensive pan-genomic approaches are needed.

## Conclusion

This chromosome-scale, phased diploid genome assembly of *Q. virginiana* represents the first high-quality genomic resource for section *Virentes*. As one of the most contiguous and complete oak genomes available, it offers new insights into the structural organization of oak centromeres, revealing patterns that warrant further investigation into their potential role in recombination, introgression, and adaptation. Our findings reinforce the longstanding hypothesis that *Quercus* genomes exhibit a high degree of synteny, particularly between *Virentes* and its sister section *Quercus*. By expanding the genomic resources available for oaks, this assembly enhances our ability to investigate the evolutionary dynamics, adaptation, and hybridization potential within *Quercus*.

## Data Availability

The raw data used to generate these assemblies and annotations is deposited on NCBI under BioProject PRJNA1310641. The assemblies for Haplotype 1 and Haplotype 2 are deposited under BioProject PRJNA1367179 and PRJNA1367180, respectively. Custom scripts for figures and analyses are available at: https://github.com/laramiemckenna/quercus_virginiana_genome. The genome assemblies and annotations as well as Supplementary data are deposited in Zenodo: https://zenodo.org/records/17674994. A table listing the available data and a way to access it is available in Supplementary Table 10. Supplementary Material is available at figshare: https://doi.org/10.25387/g3.31043098.
